# Binding interactions of *Trametes villosa* and *Trametes lactinea* laccases with 4-nonylphenol and its intermediates: molecular docking and molecular dynamics approaches

**DOI:** 10.1007/s11356-026-37475-8

**Published:** 2026-02-20

**Authors:** Robson Lourenço da Silva Santos, Nathália Felix Bomfim, Fabrício Motteran

**Affiliations:** 1 Graduate Program in Biotechnology (PPGBiotec), Department of Antibiotics, Federal University of Pernambuco (UFPE), Recife, Pernambuco, Brazil; 2 Laboratory of Environmental Sanitation, Department of Civil and Environmental Engineering, Federal University of Pernambuco (UFPE), Recife, Pernambuco, Brazil

**Keywords:** Endocrine disruptors, Bioremediation, Oxidoreductases, Biodegradation

## Abstract

Emerging pollutants such as 4-nonylphenol (4-NP) act as endocrine disruptors and have been associated with reproductive toxicity in humans and wildlife, as well as with physiological disturbances in aquatic, terrestrial, and plant organisms. Laccases are oxidoreductases with notable biotechnological relevance and the ability to oxidize phenolic pollutants, making them attractive candidates for biodegradation strategies. This study investigated the interactions between laccases from *Trametes villosa* and *Trametes lactinea* and 4-NP and its degradation intermediates via molecular docking and molecular dynamics simulations (MDS). Ligands were geometrically optimized using the PM7 semiempirical method, and their global reactivity descriptors were computed to explore correlations between electronic properties and laccase binding affinity. Docking revealed favorable binding energies (Δ*G*_bind_ ≈ −6 kcal·mol^−1^) and recurrent interactions with key amino acid residues, including Ala, Glu, Leu, Phe, Pro, Ser, Val, and His, mainly through hydrogen bonding and hydrophobic contacts. The MDS confirmed the stability of the enzyme–ligand complexes, as indicated by low root mean square deviation (RMSD) and root mean square fluctuation (RMSF) values, along with consistent radius of gyration and solvent-accessible surface areas throughout the trajectories. Binding free energy calculations using the Molecular Mechanics/Poisson–Boltzmann Surface Area (MM/PBSA) method indicated stronger binding affinity under solvation, with Δ*G*_bind_ values of −26.45 and −17.73 kcal·mol^−1^ for *T. villosa* and *T. lactinea*, respectively, highlighting hydrophobic and van der Waals contributions as the primary stabilizing forces. Overall, these results provide computational evidence that laccases from *T. villosa* and *T. lactinea* have potential for application in the oxidative biodegradation of 4-NP. These findings advance the molecular understanding of fungal laccase‒pollutant interactions and support future in vitro validation and protein engineering strategies aimed at enhancing biodegradation efficiency.

## Introduction

Population growth, urbanization, and accelerated industrialization have made environmental pollution one of the greatest challenges in recent years, leading to global concerns about water quality and food safety. The contamination of natural resources by chemical waste from industrial processes or domestic activities has a direct effect on biodiversity in terrestrial, freshwater, and marine environments, and as this contamination also represents a risk to human health, there is an increasing demand for safer and more environmentally friendly forms of remediation (Lu et al. 2015; Xiong et al. 2019; Bhandari et al. 2021).

Among environmental pollutants, those known as endocrine disruptors, most of which have complex structures such as aromatic rings, halogens, or azo groups, are persistent or recalcitrant to biological degradation and have gained interest in recent years because of their adverse effects on human health and biodiversity. The effects of exposure to this class of pollutants have been linked to endocrine disruption, resulting in reproductive disorders, retardation of neurological development, abnormal growth in children, alterations in the functions of the immune system, and the prevalence of hormone-sensitive cancers in humans (Alves da Silva et al. 2018). They are also responsible for changes in hormonal activity during the embryonic development of aquatic organisms, causing changes in anatomy, behavior, and reproduction (Chinnasamy and Poomani 2020). Endocrine disruptors, such as 4-nonylphenol, have attracted considerable attention because of their diverse applications and widespread environmental contamination (Stenholm et al. 2020).

4-Nonylphenol (C_15_H_24_O) or 4-(2,4-dimethylheptan-3-yl)phenol is an ethoxylated nonylphenol derivative characterized as a nonionic surfactant widely used in the formulation of detergents, lubricants, antistatic agents, cleaning agents for high-performance textiles, pesticides, antioxidants for rubber production, and lubricating oil additives, in addition to being used as additives in the production of plastics and UV filters in cosmetics (Beiras et al. 2019). The degradation product of ethoxylated nonylphenol, 4-nonylphenol, is listed as an environmental endocrine disruptor and a persistent organic pollutant, and the legislation regulating the permitted concentration for endocrine disruptors establishes a maximum permitted concentration for nonylphenol of 28.00 µg·L^−1^ in fresh water and 7.00 µg·L^−1^ in saltwater (US EPA 2006).

The degradation process of ethoxylated nonylphenol occurs through the loss of ethoxylated groups to form the following products: nonylphenol diethoxylate (NP2EO), nonylphenol monoethoxylate (NP1EO), nonylphenoxy ethoxy acetic acid (NP2EC), nonylphenoxy acetic acid (NP1EC), and 4-nonylphenol (4-NP). The biotransformation of nonylphenol can occur both aerobically and anaerobically; thus, under aerobic conditions, ethoxylated nonylphenol is degraded to NP2EO and NP1EO, which are immediately converted to NP2EC and NP1EC, whereas under anaerobic conditions, the conversion occurs directly to NP2EO, NP1EO, and 4-NP (Zhang et al. 2008; Ömeroǧlu and Sanin 2014).

These products are hydrophobic and more persistent and toxic than the primary parent compound, and the estrogenic activity and endocrine disrupting effects increase as the molecular weight decreases due to the degradation process (He et al. 2020). For example, in plants, exposure to 4-NP results in a decrease in chlorophyll content and stomatal size due to damage caused to the cell membrane by an increase in permeability, and in humans, some of the effects of exposure to 4-NP are associated with disturbances in the reproductive system, causing, for example, apoptosis in spermatogonial cells (Kim et al. 2019; Park et al. 2020; Gałązka and Jankiewicz 2022).

In this context, there has been an increasing search for efficient and viable alternatives for the degradation of this contaminant. Laccases, enzymes of great biotechnological importance, exhibit a strong ability to oxidize phenolic compounds (Catherine et al. 2016). This group of enzymes is widely distributed in Basidiomycota and can also be found in bacteria and even crustaceans (Strong and Claus 2011; Shi et al. 2017; Beck et al. 2018). Laccases belong to the class of copper-containing polyphenol oxidases and typically possess four copper atoms that constitute the active site region. These enzymes are characterized by broad substrate specificity, with catalytic activity occurring through the oxidation of an electron from the substrate at the type 1 copper site, which acts as the primary electron acceptor (Orlando et al. 2024). This process is followed by the transfer of four electrons to molecular oxygen, resulting in its reduction to two water molecules (Lloret et al. 2010; Solomon et al. 2014). Notably, this reaction does not require additional cofactors, and water is the only by-product, highlighting laccases as promising biocatalysts for pollution abatement (Wang et al. 2024).

Laccases have a wide range of applications, including in the food industry for baking and juice processing, wine stabilization, beer preservation, and wastewater bioremediation; in the textile industry for dye removal from effluents and fabric bleaching; and in nanobiotechnology for the detection of molecules and compounds such as polyphenols and for biosensor development (Khatami et al. 2022). Although fungal remediation of several phenolic pollutants has been associated with laccase activity in species of the *Trametes* genus, existing studies usually focus on degradation efficiency and the identification of transformation intermediates (Mo et al. 2018; Mohit et al. 2019). However, detailed analyses addressing enzyme–ligand interaction mechanisms, particularly at the molecular level and involving 4-nonylphenol and its degradation intermediates, have not been explored in the literature.

In addition, *Trametes villosa* and *Trametes lactinea* are fungal species belonging to the phylum Basidiomycota and the family Polyporaceae. These species are widely distributed worldwide and are representatives of white-rot fungi, being capable of decomposing all major components of wood (Tomé et al. 2022; Dong et al. 2024; Maldonado-Pérez et al. 2025). Recently studied research reveals that these species have potential for degrading environmental pollutants such as textile industry dyes; this degradation potential is related to the presence of laccases; however, there are no studies on the ability of laccases from these species to degrade 4-nonylphenol (Ferreira-Silva et al. 2022).

Therefore, the aim of this study was to evaluate the potential of laccases produced by *T. villosa* and *T. lactinea* for the degradation of the endocrine disruptor 4-nonylphenol and its intermediates in terms of binding affinity. Overall, this study provides mechanistic insights into laccase-mediated nonylphenol degradation and indicates the potential of laccases from *Trametes villosa* and *Trametes lactinea* for future exploration in environmentally sustainable bioremediation strategies and the rational design of enzymatic systems for pollutant removal.

## Materials and methods

In this study, an integrated computational approach was employed to investigate the interaction between laccases from *Trametes villosa* and *Trametes lactinea* and 4-nonylphenol (4-NP) and its main intermediates. First, the molecular structures of 4-NP and its intermediate products were optimized geometrically using classical and semi-empirical quantum methods. Then, thermodynamic and global reactivity descriptors were calculated to assess their electronic properties and reactivity profiles. Meanwhile, three-dimensional models of the laccases were constructed via homology modeling and validated to ensure structural reliability. Then, molecular docking simulations were performed to predict the binding modes, interaction patterns, and affinities of the enzymes and ligands. Finally, molecular dynamics simulations were conducted to evaluate the stability, conformational behavior, and energetic profile of the most favorable enzyme–ligand complexes over time. This included binding free energy estimation using the MM/PBSA method. This multistep workflow enabled a comprehensive evaluation of the structure–reactivity–binding relationships underlying laccase-mediated interactions with 4-NP.

### Geometric optimization of 4-NP and NP2EC, NP1EC, NP2EO, and NP1EO intermediates

The aim of geometric optimization is to determine the most stable three-dimensional molecular structure by minimizing the system’s potential energy as a function of atomic coordinates. Classical force-field methods efficiently provide an initial approximation of molecular geometry, while semiempirical quantum mechanical methods further refine the structure by incorporating electronic effects. This combined approach achieves a reliable balance between computational efficiency and accuracy, generating optimized conformations suitable for subsequent electronic property calculations and molecular interaction studies (Gieseking 2021).

The three-dimensional structures of 4-NP and its intermediates were obtained from the PubChem database of the National Center for Biotechnology Information (NCBI) (Kim et al. 2021), and the corresponding PubChem CID codes are 1752 (4-NP), 18,380 (NP1EC), 86,267 (NP2EC), 154,827,466 (NP1EO), and 24,773 (NP2EO). The structures of the nonylphenols were preoptimized via the classical MMFF94s method via Avogadro software version 1.2.0, and then, the structures were reoptimized via the PM7 quantum method via MOPAC2016.

### Thermodynamic and global reactivity descriptors

In MOPAC2016, the energies of the formation enthalpy (∆Hf^0^) and of the HOMO and LUMO frontier orbitals were calculated. To correlate the reactivity/stability of these molecules with their binding affinity to laccase, the GAP energies, which are the difference between the LUMO and HOMO energies (Eq. 1), were subsequently determined (Kamel and Mohammadifard 2021; Feizi-Dehnayebi et al. 2021). The HOMO and LUMO energies were also used to obtain other properties that are important indicators of reactivity. These parameters are the electronic affinity (*A*), ionization potential (*I*), absolute hardness (*η*), softness (*σ*), electronegativity (*χ*), electrophilicity (*ω*), chemical potential (*μ*), and nucleophilicity (*N*), and the corresponding equations for calculating each parameter are found below (Pilli et al. 2015; Miar et al. 2021).

The correlations between the theoretical descriptors and binding energies obtained from docking were evaluated via Pearson’s correlation coefficient. Statistical significance was set at *p* < 0.05. All analyses were performed via GraphPad Prism (version 10). The strength of positive and negative correlations was interpreted according to Akoglu (2018) as follows: perfect (*r *= 1.0 or –1.0), very strong (|*r*| > 0.8), strong (0.6 < |*r*| ≤ 0.8), moderate (0.3 < |*r*| ≤ 0.6), weak (0.1 < |*r*| ≤ 0.3), and negligible (|*r*| ≤ 0.1). Owing to the limited number of ligands (*n* = 5), the correlation analysis was interpreted as exploratory, aiming to identify trends rather than infer statistical causality.

1 $$GAP:\;Eg\;\left(\varepsilon LUMO-\varepsilon HOMO\right)$$

2 $$Electronic\;affinity:\;A=-\left[\varepsilon LUMO\right]$$

3 $$Ionization\;potential:\;I=-\left[\varepsilon HOMO\right]$$

5 $$\begin{array}{c}Absolute\;hardness:\eta=\frac{\left(\varepsilon LUMO-\varepsilon HOMO\right)}2\\or\\\eta=\frac{\left(I-A\right)}2\end{array}$$

6 $$Softness:\;\sigma=\frac1\eta$$

7 $$\begin{array}{c}Electronegativity:\;\chi=-\frac{\left(\varepsilon LUMO+\varepsilon HOMO\right)}2\\or\\x=\frac{\left(I+A\right)}2\\or\;\\x=-\mu\end{array}$$

8 $$Electrophicility:\omega=\frac{x^2}{2\eta}$$

9 $$Nucleophicility:N=\frac1\omega$$

### Homology modeling of *Trametes villosa* and *Trametes lactinea* laccases

Homology modeling is one of the most commonly used methods for obtaining three-dimensional structures of biological macromolecules. This technique is based on the principle that sequence similarity implies structural and, in many cases, functional similarity. Accordingly, protein homology modeling uses experimentally determined template structures to predict the three-dimensional conformation of a target protein based on the similarity of their primary amino acid sequences (Agnihotry et al. 2022).

There are currently no crystallographic structures available in the RCSB Protein Data Bank (PDB) for the laccase enzymes from *Trametes villosa* and *Trametes lactinea*. Therefore, their three-dimensional structures were generated through homology modeling based on the primary sequences (FASTA codes AAC41687.1 and KAH9897825.1) retrieved from the NCBI GenBank database.

The models were built via the online Swiss-Model platform, employing the crystal structures 5A7E (for* T. villosa*) and 2HRG (for *T. lactinea*) as templates. Model validation was performed via the MolProbity score and Ramachandran plot provided by SWISS-MODEL, as well as the VERIFY 3D and ERRAT tools available at https://saves.mbi.ucla.edu/, which assess stereochemical quality and overall model reliability (Arnold et al. 2006).

### Molecular docking simulation

The molecular docking method provides detailed insights into the recognition and binding modes of protein–protein and receptor–ligand interactions. Its primary purpose is to accurately predict the geometry of a target compound that best fits a specific enzyme’s binding site (Renuga Parameswari et al. 2015). This allows the estimation of the most probable binding affinity under realistic conditions and provides fundamental physicochemical information that elucidates the intermolecular interactions between proteins, enzymes, and ligands (Manogar et al. 2019).

The first step in this phase of the study was to prepare the receptors (laccase from *T. villosa* (LacTv) and laccase from *T. lactinea* (LacTl)) for the docking simulation, which consisted of removing all the water molecules and adding the polar hydrogens and Kollman charges (Patil et al. 2021), using the software Biovia Discovery Studio v.20.1.0.19295 and AutoDock Tools v.1.e5.6.

The docking parameters were then applied, allowing semiflexible docking in which all the ligand torsional bonds were free to rotate while the receptor was kept rigid. A 1 Å grid box was centered on the T1 copper ion, with coordinates and dimensions adjusted to include the T2, T3α, and T3β sites within the generated grid area.

The molecular docking simulation was configured via the Lamarckian genetic algorithm. The resulting .pdbqt files were then generated and submitted to AutoDock Vina v1.1.2, with an exhaustiveness value of 64 (Che et al. 2023). Among the nine conformations generated by AutoDock Vina, the first conformation, with zero root mean square deviation (RMSD) and the lowest free energy, was used to visualize the molecular interactions of the receptor‒ligand complex via Discovery Studio Visualizer v.20.1.0.19295 software (Patil et al. 2022).

### Molecular dynamics simulation

Molecular dynamics simulations (MDS) comprise a set of tools that allow the study of the behavior of systems of many particles over a predetermined time. These simulations enable an understanding of the physical basis of the structure and function of biological macromolecules (Grewal et al. 2025). MDS techniques provide information on the microscopic dynamic behavior of each atom in a system. This allows for the simulation and analysis of their movements, interactions, and properties at the atomic level. The goal is to obtain observable properties such as temperature, pressure, internal energy, volume, interaction energy, entropy, and free energy (Namba et al. 2008). In environmental contaminant degradation studies, MDS techniques are used to explore interactions between enzymes and pollutant molecules. These techniques provide high-precision data on the stability, dynamics, and energetics of enzyme-ligand complexes, facilitating an understanding of contaminant degradation processes and the development of environmental remediation strategies (Hoang et al. 2024).

The MDS for the laccase-4-NP complexes generated by docking was performed via the GROMACS 2023.3 package. The topology for the laccases of both species was obtained via the charmm36 force field, and CHARMM-CGenFF was used to generate the .str file of the 4-NP topology via the ligand file extracted from the complex obtained by docking in the .mol2 format. The complexes were centralized in a dodecahedral box with 9.024 nm boundaries; the boxes were then solvated via the TIP3P water model, and Cl^−^ and Na^+^ ions were added to neutralize the system. System energies were minimized via the steepest descent algorithm to reduce the occurrence of unfavorable interactions. Equilibrium simulations were performed for 1 ns under NVT and NPT conditions using a V-rescale thermostat with a target temperature of 300 K and a Berendsen barostat with a target pressure of 1 bar. To produce the MDS of the systems in equilibrium, a time of 1 ns was adopted for the analyses (Kwiatos et al. 2020; Sharma et al. 2020), and the script was configured to save the energies and trajectory coordinates every 1 ps. The Particle Mesh Ewald (PME) method was used to treat long-distance electrostatic interactions, and the Verlet method was used for short-distance interactions.

To analyze the lacase–4-NP complex stability during the dynamics simulation time, the temperature variation was evaluated via NVT simulation, the density and pressure during the equilibration of the system were evaluated via NPT simulation, and the radius of gyration, the protein–ligand complex RMSD, and the root mean square fluctuation (RMSF) of the protein were evaluated during the MDS in the equilibrated system. To analyze the lacase-4-NP interaction, plots of the interaction energies and the number of hydrogen bonds occurring between lacase and 4-NP during the dynamics were plotted, and the protein‒ligand binding free energies (∆*G*_bind_) were calculated via the Molecular Mechanics/Poisson–Boltzmann Surface Area (MM/PBSA) method based on the basis of the molecular dynamics trajectory generated by gromacs.

## Results and discussion

### Geometric optimization and thermodynamic and reactivity descriptors

The MMFF94s force field was used to preoptimize the crystals, as this is the most appropriate method for this type of study, as it has been shown to provide good accuracy in minimizing energy in a variety of organic molecules and bioactive compounds (Wahl et al. 2019). The resulting structure superimposed on the structure obtained from PubChem showed an acceptable RMSD of 0.0984 Å for 4-NP, so the same method was used for energy optimization of all nonylphenols (Novič et al. 2016).

Semiempirical methods, on the other hand, allow the user to obtain other conformations of the compound by exercising a set of assumed energies for each atom in the molecule, simulating a set of experimental data such as equilibrium geometries, heats of formation, dipole moments, and ionization energies (Sant’Anna 2009). The RMSD obtained via the PM7 method was 0.1079 Å. The PM7 method was chosen for the final optimization of nonylphenols because it is a suitable method for modeling enzyme-catalyzed reactions. Compared with other semiempirical methods, it has been shown to provide more accurate results in terms of the enthalpy of formation, proving to be very efficient in predicting the interaction of ligands in protein environments (Stewart 2017). These results demonstrate that the methodology employed exhibits a degree of accuracy comparable to that of the procedures utilized for the structures available in the PubChem database.

The frontier orbitals HOMO (highest occupied molecular orbital) and LUMO (lowest unoccupied molecular orbital) are often considered to be the most important frontier orbitals because they are able to determine how a molecule behaves when interacting with other species, since the electronic affinity (A) of a molecule is given by the energy of the LUMO and the ionization potential (I) is given by the energy of the HOMO (Pilli et al. 2015).

The GAP calculation (Table 1) shows these energies and indicates that the smaller the GAP between the energies of the orbitals is, the more reactive and polarizable the molecule, whereas higher GAP values indicate greater stability of the molecules. This relationship is related to the concepts of hardness and softness in such a way that the higher the GAP between the energies of these orbitals is, the greater the hardness and the lower the softness (Zhang and Musgrave 2007; Karunakaran and Balachandran 2012).

**Table 1:** Global reactivity descriptors for 4NP and intermediates

Descriptors	4NP	NP1EC	NP1EO	NP2EC	NP2EO
Enthalpy of formation (∆Hf^0^) (kJ mol^−1^)	−332.30	−681.85	−514.14	−866.86	−692.51
HOMO (eV)	−9.18	−9.22	−9.06	−9.08	−8.98
LUMO (eV)	−0.11	−0.15	−0.06	−0.08	−0.02
LUMO–HOMO gap (eV)	9.07	9.07	9.00	9.00	8.97
Ionization potential (*I*) (eV)	9.18	9.22	9.06	9.08	8.98
Electronic affinity (*A*) (eV)	0.11	0.15	0.06	0.08	0.02
Electronegativity (*χ*)	4.65	4.68	4.56	4.58	4.50
Chemical potential (*μ*)	−4.65	−4.68	−4.56	−4.58	−4.50
Absolute hardness (*η*)	4.53	4.53	4.50	4.50	4.48
Softness (*σ*)	0.22	0.22	0.22	0.22	0.22
Electrophilicity (*ω*)	2.38	2.42	2.31	2.33	2.26
Nucleophilicity (*N*)	0.42	0.41	0.43	0.43	0.44

The results obtained by calculating the GAP increased with the degradation of ethoxylated nonylphenol, whether for compounds formed by aerobic or anaerobic biodegradation, with GAP values of 9.00, 9.07, and 9.07 for NP2EC, NP1EC, and 4-NP, respectively, and GAP values of 8.97 and 9.00 for NP2EO and NP1EO, respectively. The high GAP value reflects the high stability of these compounds, and the increase in these values with decreasing ethoxylated groups confirms greater recalcitrance and difficulty in degrading the final compound 4-NP in nature compared with its precursors (Bergé et al. 2012) and emphasizes the need to reduce the risks represented by emerging contaminants such as nonylphenol to the environment and biodiversity, as the results also show high values of electrophilicity, indicating the potential toxicity of the compounds (Chinnasamy and Poomani 2020).

### Homology modeling of laccase

**Fig. 1:**
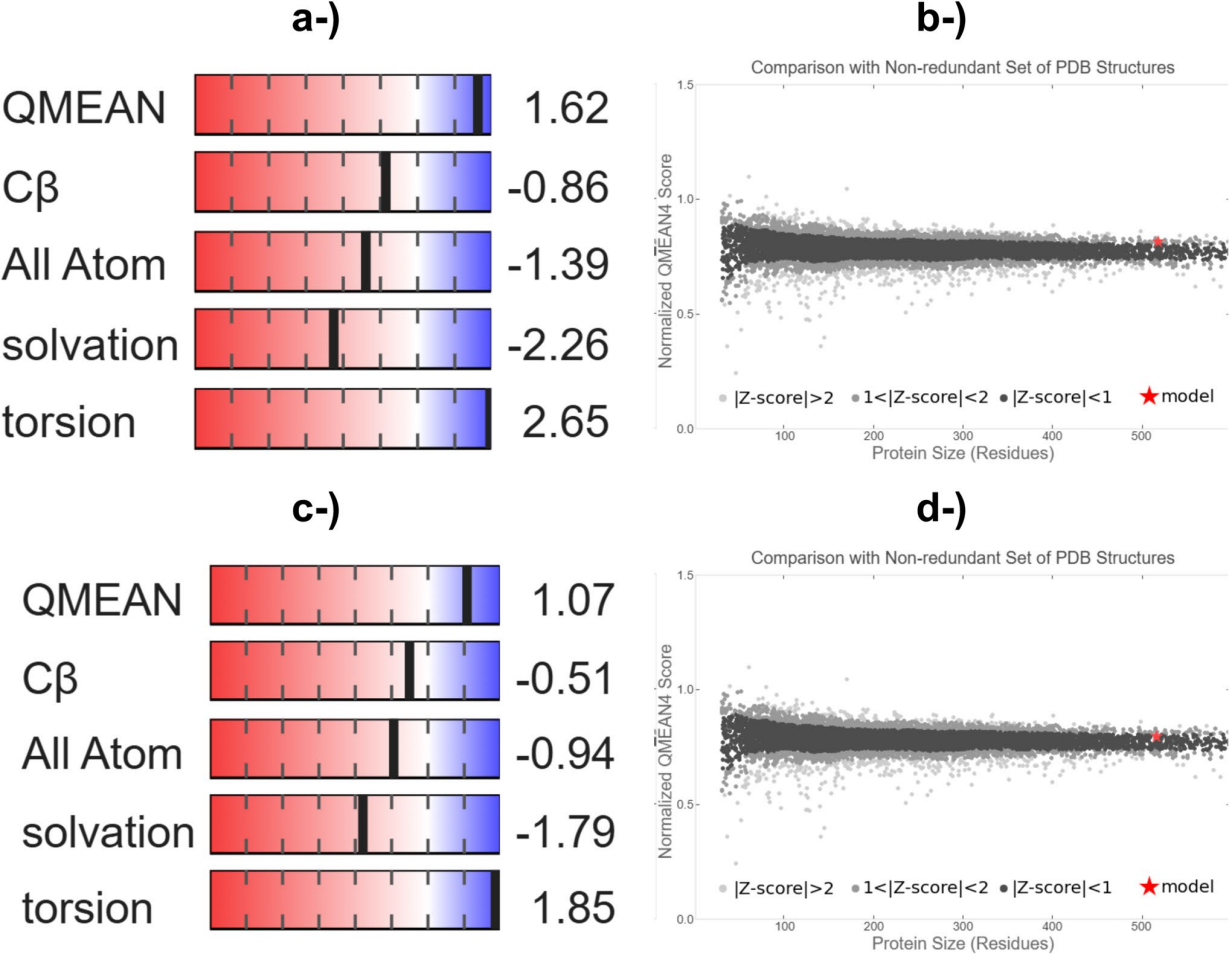
Estimation of model quality predicted by the QMEAN calculation for the *T. villosa* laccase model (**a**) and its corresponding normalized *Z* score (**b**) generated using SWISS-MODEL. Likewise, panels **c** and **d** represent the QMEAN quality estimation and normalized *Z* score for the *T. lactinea* laccase model, respectively. The blue gradient indicates better alignment between the target and template structures, while the red gradient indicates poorer alignment

The models showed excellent GMQE values (Fig. 1), indicating a high reliability index with values very close to 1, and satisfactory QMEAN values, below 2, indicating a *Z* score with good QMEAN normalization and a high degree of natality and consistency of the model’s interatomic distances compared with experimentally determined homologous protein information (Waterhouse et al. 2018).

The results obtained from the *Errat* and *Verify* analyses validated the quality of the models, indicating average overall quality factors of 92.84 for LacTv and 90.98 for LacTl. These values are considered satisfactory, as *Errat* scores above 80% are indicative of reliable models. Similarly, the *Verify* assessment yielded values of 93.59 for LacTv and 87.30 for LacTl, suggesting that the majority of residues occupy energetically stable and favorable positions within the models (Román et al. 2020).

**Fig. 2:**
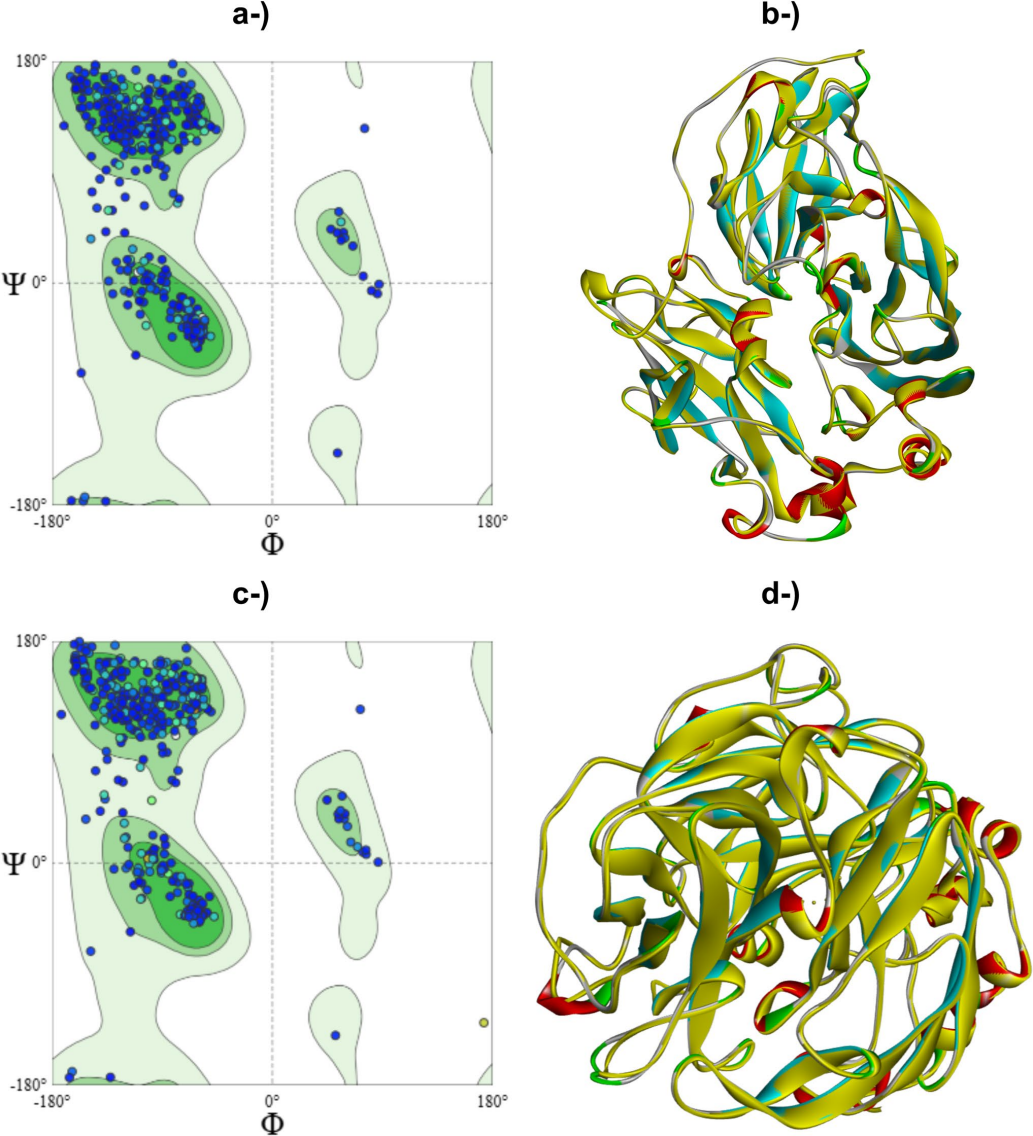
Ramachandran plots of homology models generated by SWISS-MODEL for LacTv (**a**) and LacTl (**c**); the plots display the distribution of backbone dihedral angles (*ϕ* and *ψ*) for amino acids’ residues of the models. The residues are predominantly located within the favored (dark green) and additionally allowed (light green) regions, which indicates the predicted models have good stereochemical quality and structural reliability. The model of laccase from *T. villosa* superimposed on template 5A7E (PDB) of laccase from *Coriolopsis gallica* (**b**) and the model of laccase from *T. lactinea* superimposed on template 2HRG (PDB) of laccase from Trametes trogii (**d**); the models obtained are highlighted in yellow

The models also showed favorable results for structural validation, both by the Ramachandran map and MolProbity score (Singh et al. 2025). The percentages of residues located in favorable regions were 96.38 and 95.75% (Fig. 2a and c). The MolProbity scores obtained were 1.41 (LacTv) and 1.37 (LacTl), indicating good structural quality for both models. These results validate the models obtained for the laccases of both species, since a good model is expected to have 90% or more residues in favorable regions (Gopalakrishnan et al. 2007; Carrascoza et al. 2014; Chandrasekaran et al. 2017). The superposition of the models that best fit the template used for modeling is shown in Fig. 2.

### Molecular docking and interaction mechanisms

The binding free energy between the ligands 4-NP, NP1EC, NP1EO, NP2EC, and NP2EO and laccase (Table 2) predicted in the molecular docking simulation shows a binding affinity with LacTv values under −5.0 kcal·mol^−1^, with a ∆*G*_bind_ of −5.9 kcal·mol^−1^ for the interaction with 4-NP, and values lower than −5.5 kcal·mol^−1^ for LacTl, with a ∆*G*_bind_ of −6.2 kcal·mol^−1^ for the interaction with 4-NP. Negative ∆*G*_bind_ values are considered favorable for degradation, so the results obtained by docking suggest that the degradation of 4-NP and its intermediates by LacTv and LacTl is spontaneous. In addition, the affinity between the laccases of both species and 4-NP is the strongest among the ligands studied, with the most negative ∆*G*_bind_ values of approximately −6.0 kcal·mol^−1^ and −6.2 kcal·mol^−1^ with LacTv and LacTl, respectively, which indicates that, compared with their intermediates, 4-NP can be more easily degraded by LacTv and LacTl, since the lower the enzyme–substrate binding energy is, the more stable the interaction and the easier the contaminant complexation and degradation (Hongyan et al. 2019).

**Table 2:** Free binding energy (theoretical ∆*G*_bind_ in kcal·mol^−1^) of LacTv and LacTl with 4NP and its intermediates

Ligands	∆*G*_bind_ (kcal·mol^−1^)	
	*Trametes villosa*	*Trametes lactinea*
4-NP	−5.9	−6.2
NP1EC	−5.5	−5.7
NP1EO	−5.1	−5.5
NP2EC	−5.0	−5.8
NP2EO	−5.4	−5.5

**Fig. 3:**
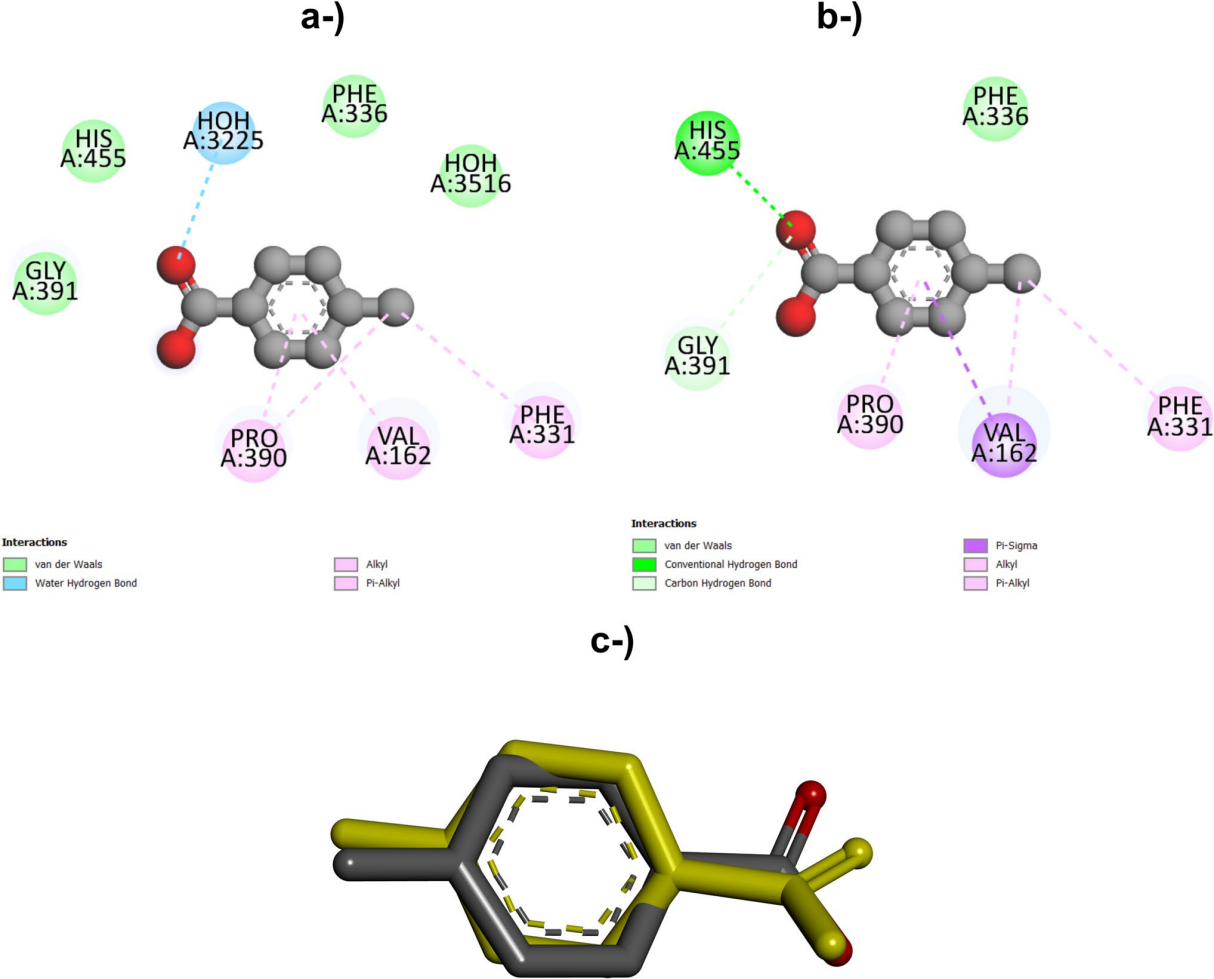
Redocking of *T. trogii* laccase–methyl benzoate complex (PDB ID: 2HRG). Interactions observed for the crystallized laccase–methyl benzoate complex determined experimentally (**a**). Interactions identified through redocking of the methyl benzoate–laccase complex (**b**). Superposition of the lowest-energy redocked pose with the experimentally determined structure of the *T. trogii* laccase p-methyl benzoate complex (PDB ID: 2HRG)

The redocking of the p-methylbenzoate ligand with *T. trogii* laccase to the crystal 2HRG complex obtained from the PDB resulted in an RMSD of 1.2176 Å, demonstrating that the protocol used is able to reproduce the position of the ligand in the active binding site and predict the same amino acid residues of the protein that interact with the ligand, as observed experimentally (Fig. 3). The free binding energy of the 4-NP–2HRG interaction was −5.9 kcal·mol^−1^, a result very similar to that obtained for the docking of nonylphenols with LacTv and LacTl.

Therefore, these results validate the docking protocol used in this work. Since validation by redocking requires RMSD values lower than 2.0 Å, the results obtained for docking with laccase via homology modeling are similar to the results obtained for docking with a known laccase determined experimentally (Hevener et al. 2009; Karunakaran and Muniyan 2023).

**Fig. 4:**
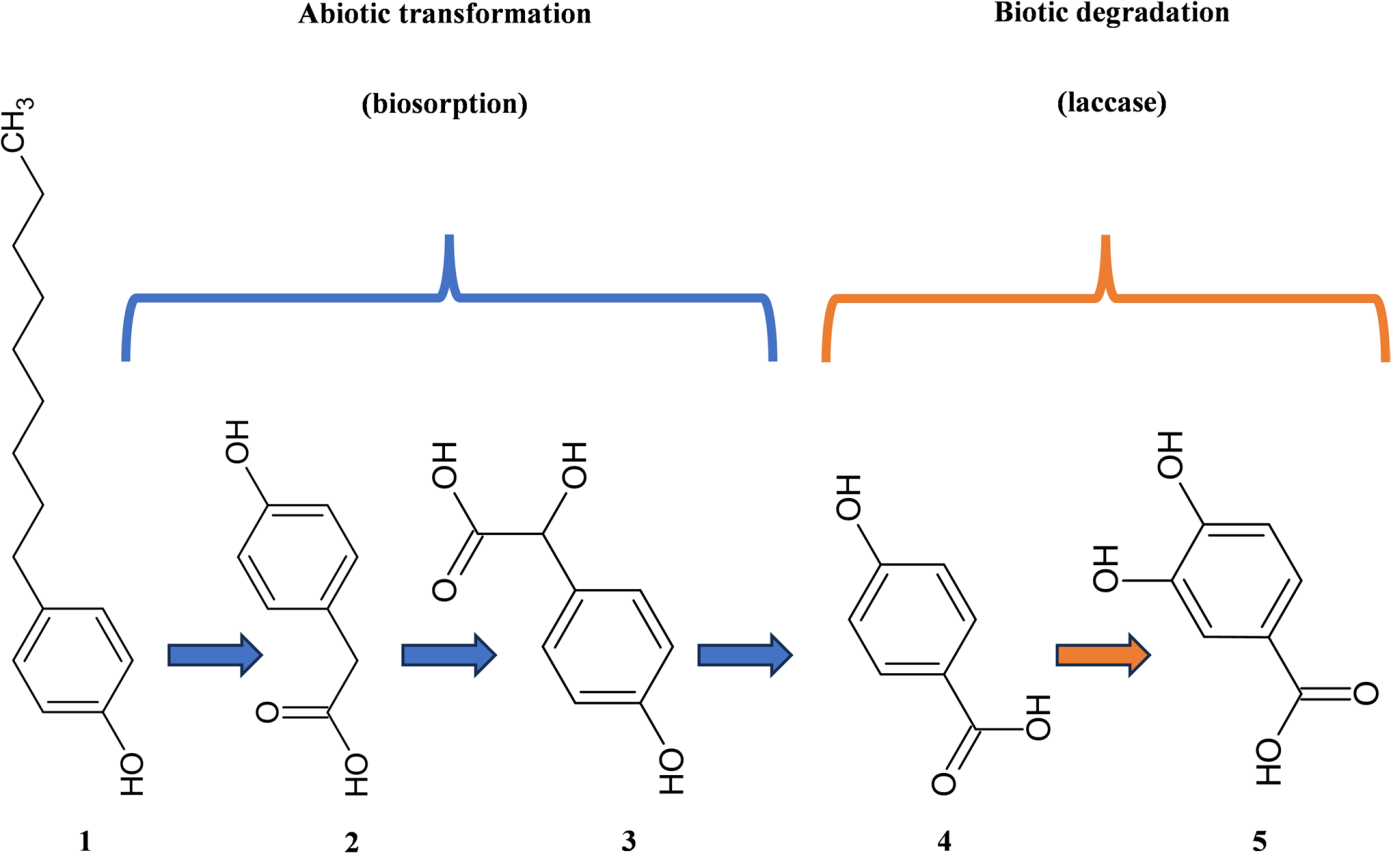
Proposed degradation pathway of the 4NF (1) isomer with a linear side chain by the fungus *Thielavia* sp., as described by Mtibaà et al. (2020). The process involves an abiotic transformation (blue arrows), mainly through biosorption, followed by a biotic degradation step catalyzed by laccase (orange arrow). The intermediates involved are 4-hydroxyphenylacetic acid (2), 2-hydroxy-2-(4-hydroxyphenyl)acetic acid (3), 4-hydroxybenzoic acid (4), and 3,4-dihydroxybenzoic acid (5) Source: Adapted from Mtibaà et al. (2020) and drawn by the author using ChemSketch

Some studies have reported the involvement of fungal laccases in the degradation of 4-NP, but there is no report with detailed information on the metabolic pathway and degradation products specifically formed by the action of this enzyme (Catapane et al. 2013; Garcia-Morales et al. 2015; Spina et al. 2015; Catherine et al. 2016; Mallerman et al. 2019; Stenholm et al. 2020; Gałązka and Jankiewicz 2022). In addition, most of the studies found did not evaluate the action of laccase alone but rather fungal remediation. This involves other oxidoreductase enzymes, such as peroxidases, making it difficult to establish a pathway for the degradation of 4-NP by laccases, as other enzymes are involved in the formation of the products. On the other hand, studies of laccase activity with 4-NP have been limited to evaluating the reduction in the estrogenic activity of the compound or the elimination rate (Catapane et al. 2013; Spina et al. 2015; Mallerman et al. 2019), and when the formation of products was detected, they were not identified (Garcia-Morales et al. 2015). In a study of 4-NP degradation by laccase-producing fungi, 4-NP degradation products were identified via LC–MS analysis. However, seven of them were found to be associated with abiotic transformation, with 3,4-dihydroxybenzoic acid being the only one of the identified compounds associated with the presence of laccase, suggesting the involvement of this enzyme in the final stage of degradation (Fig. 4) (Mtibaà et al. 2020).

Although the role of laccase in the degradation of 4-NP has not yet been elucidated, it is evident that the fungal degradation of this compound occurs by shortening the alkyl chain with the consequent formation of 3,4-dihydroxybenzoic acid, which is the last product formed before the cleavage of the aromatic ring (Rózalska et al. 2015; Mtibaà et al. 2020). Degradation of 4-NP can occur, for example, by hydroxylation, mainly in the alkyl chain, but oxidative attack can also occur with hydroxylation of the aromatic ring in nonylphenol isomers with highly branched alkyl chains (Mtibaà et al. 2020). The interactions of the amino acids in the active site of LacTv and LaTl with 4-NP and its intermediates and the possible interactions in the alkyl chain and aromatic ring are presented and discussed in the next sections.

#### LacTv-nonylphenol docking

**Fig. 5:**
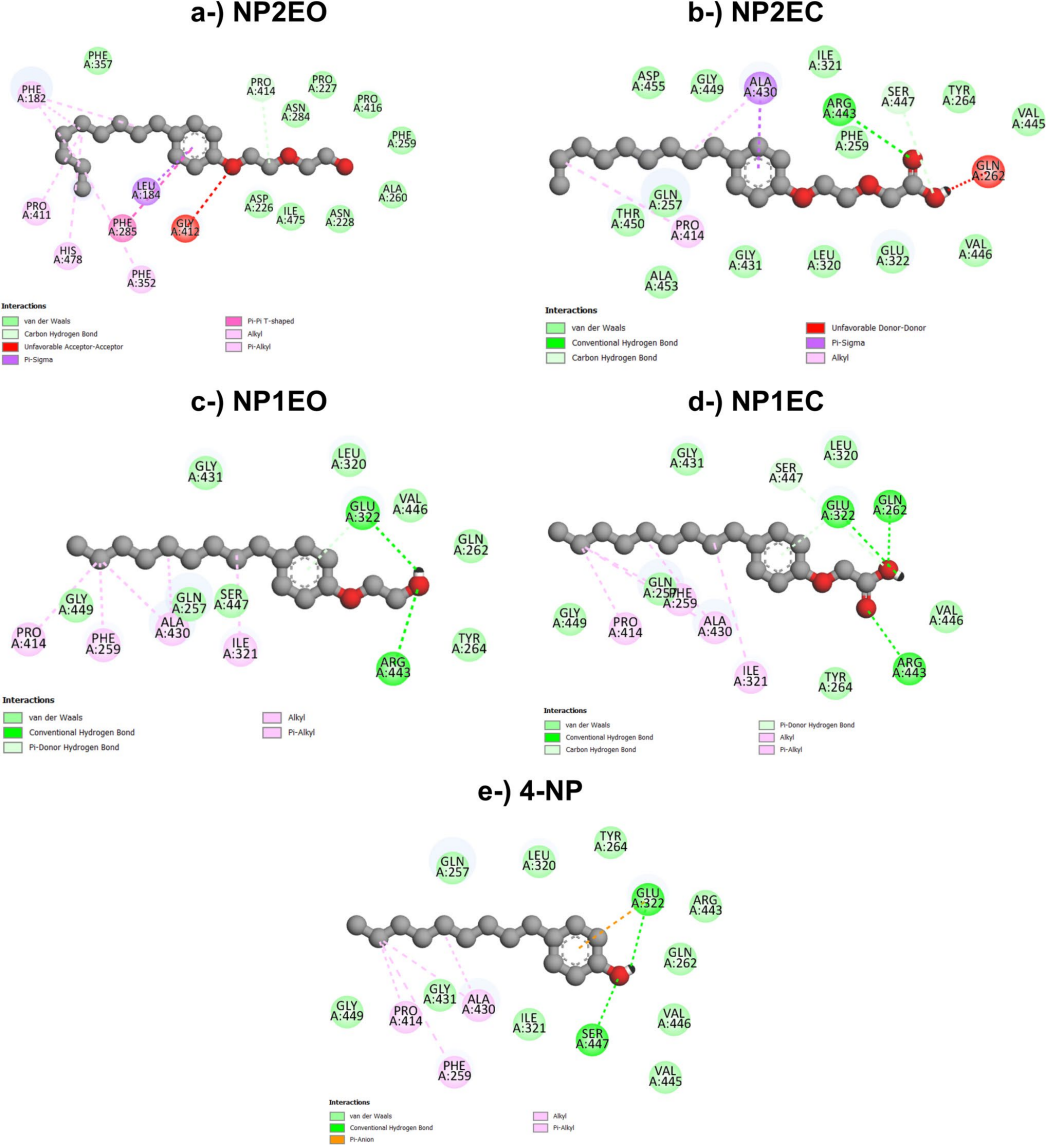
2D representations of the interactions between amino acid residues at the oxidative site of *T. villosa* laccase (LacTv) with the compounds NP2EO (**a**) and NP1EO (**c**), observed during the degradation of nonylphenol ethoxylates under both aerobic and anaerobic conditions. Figures **b** and **d** illustrate the interactions of the oxidative site residues with NP2EC and NP1EC, respectively, which occur exclusively under aerobic conditions. Figure **e** shows the 2D interactions of the oxidative site residues of LacTv with 4-NP

The molecular docking results show that LacTv interacts with 4-NP and its intermediates via hydrogen bonds, carbon‒hydrogen bonds, unfavorable donor‒donor, unfavorable acceptor‒acceptor, π‒anion, π‒sigma, T-shaped π‒π, π‒alkyl, alkyl, carbon, and van der Waals bonds. The amino acids Ala430, Arg443, Gln262, Glu322, Gly412, His478, Ile321, Leu184, Phe182, Phe259, Phe352, Pro411, Pro414, and Ser447 are suggested by the docking analysis to be the central residues involved in the interaction of LacTv with 4-NP, NP1EC, NP1EO, NP2EC, and NP2EO. The LacTv–4-NP complex is shown in Fig. 5.

**Fig. 6:**
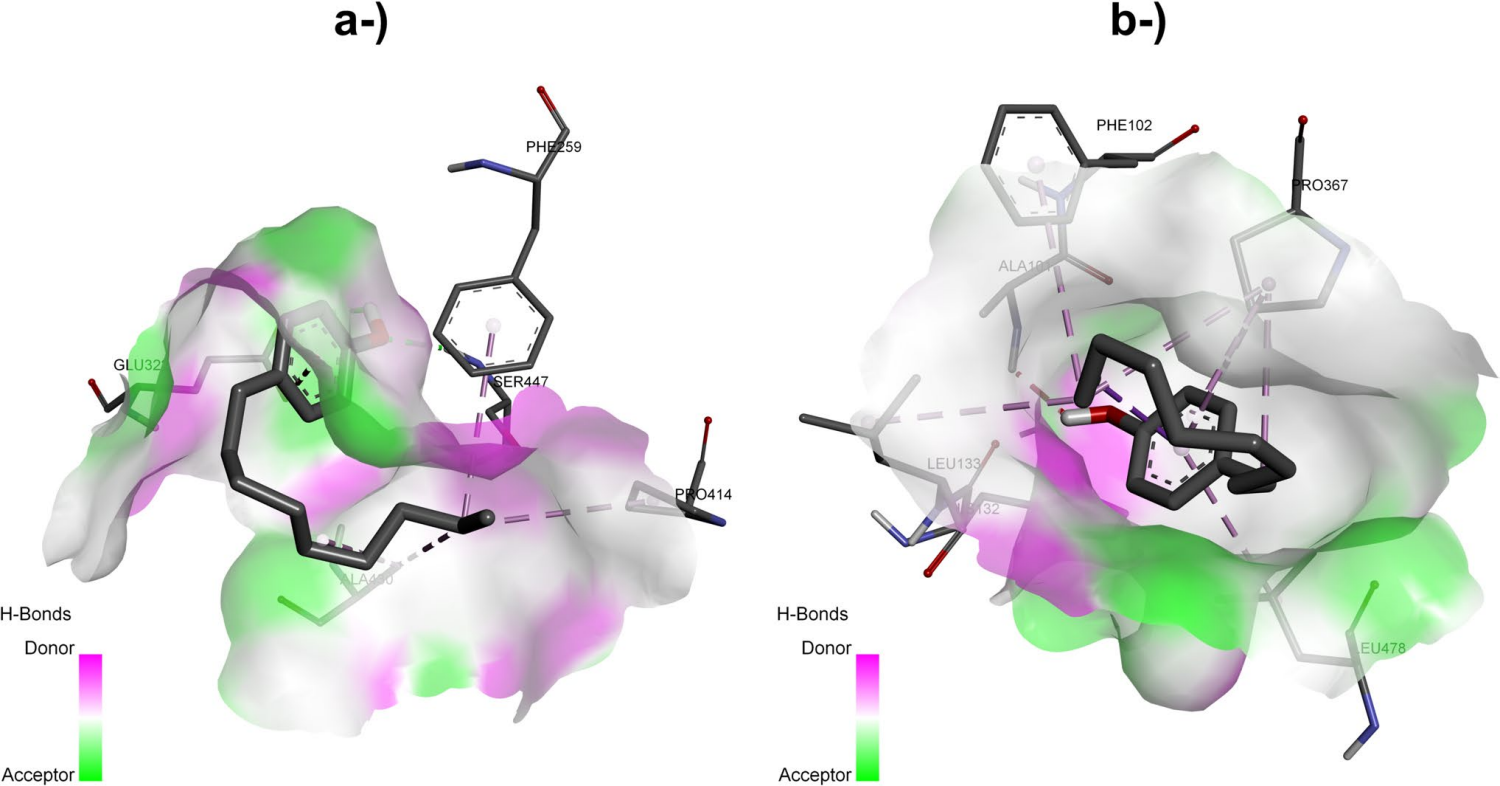
3D diagrams of molecular interactions and contact surfaces between 4-NP and *T. villosa* laccase (LacTv) (**a**) and *T. lactinea* laccase (LacTl) (**b**), generated using Discovery Studio

Glu322 plays an important role in the stability of the complexes formed between LacTv-4-NP and LacTv-intermediates through hydrogen bonds between the oxygen of its side chain in the donor and acceptor regions of the laccase binding site and the hydroxyl groups of 4-NP, NP1EC, and NP1EO; the π-anion bond of the second oxygen of the side chain with the aromatic ring of 4-NP (Fig. 6a); the π-donor hydrogen bond between one of the hydrogens of the amino group and the aromatic ring of NP1EC and NP1EO; and van der Waals interactions with NP2EC.

The interactions of Arg443 in the complexation mechanism predicted by the docking of LacTv with 4-NP and its intermediates are due to the formation of hydrogen bridges with the carbonyls of NP1EC and NP2EC and with the hydroxyl of NP1EO and van der Waals interactions with 4-NP.

The presence of negatively or positively charged polar amino acids such as Arg443 and Glu322 contributes to the formation of hydrogen bonds, as they have nitrogen and oxygen with available electron pairs. In addition, arginine is often considered the most hydrophilic amino acid because of the presence of a guanidino group in its side chain, which allows it to form up to six hydrogen bonds (Hristova and Wimley 2011; Kadam et al. 2018; Mehra et al. 2018).

His478 residue participates in the formation of the laccase–NP2EO complex through hydrophobic π–alkyl bonds. The aromatic amino acids Phe182, Phe259, and Phe352 also participate through the same type of interaction, forming bonds between Phe182 and NP2EO; Phe352 and NP2EO; and Phe259 with 4-NP, NP1EC, and NP1EO. The amino acid Phe259 also has van der Waals interactions with NP2EC and NP2EO, and Phe285 forms a π–π T-shaped bond between the aromatic side chain and the aromatic ring of NP2EO.

Interactions with uncharged polar amino acids such as Gln262 and Ser447 occurred through the formation of hydrogen bridges between Gln262 and NP1EC, unfavorable donor–donor bonds between Gln262 and NP2EC, and van der Waals interactions of Gln262 with 4-NP and NP1EO. The amino acid Ser447 forms a hydrogen bond between a hydrogen of the amine group and the oxygen of 4-NP, a side-chain carbon bond with the hydroxyl of NP1EC, a carbon–hydrogen interaction with NP2EC, and a van der Waals interaction with NP1EO.

Interactions of the ligands with the apolar amino acids of LacTv occurred between residues Ala430, Gly414, Ile321, Leu184, Pro414, and Pro411. π‒sigma bonds occurred between Ala430-NP2EC and Leu184-NP2EO and were attributed to the interaction of the aromatic ring of both ligands with the side chain of the amino acids. Alkyl-type bonds in the region of the ligand apolar chain with the amino acid side chain were formed between Ala430 and the ligands 4-NP, NP1EO, NP2EC, and NP1EC; Ile221 and the ligands NP1EO and NP1EC; Pro414 and the ligands 4-NP, NP1EC, NP1EO, and NP2EC; and Pro411 and NP2EO. Ile321 also forms a weak interaction with 4-NP and NP2EC, and Pro414 forms a carbon‒hydrogen bond with carbon 3 of NP2EO.

The alkyl and π-alkyl interactions formed between Phe259, Ala430, and Pro414 and 4-NP may have contributed substantially to the shortening of the ligand alkyl chain for the formation of 3,4-dihydroxybenzoic acid, identified by Mtibaà et al. (2020) as a product of the degradation of 4-NP by laccase-producing fungi.

The formation of hydrogen bonds with the amino acids Glu322, Ser447, Gln262, and Arg443, mainly with the hydroxyls of the phenolic groups, suggests that these residues are crucial in the degradation mechanism of the intermediates NP1EC, NP1EO, NP2EC, and 4-NP. Hydrogen bonds are responsible for the strongest intermolecular interactions among noncovalent bonds and are therefore responsible for the bioactive conformations of the macromolecule and for maintaining the stability of the complex formed between the enzyme and the ligand (Xue et al. 2023). In addition, hydrogen bonds, along with other noncovalent bonds between the protein and the ligand, are essential for maintaining the target molecule within the active site (Dunn 2010; Oliveira 2015).

Amino acid residues with aromatic side chains, such as Phe182, Phe259, Phe352, and Phe285, and hydrophobic side chains, such as Pro414, contribute to the interaction with phenolic compounds and are involved in π–π stacking, π–alkyl, alkyl, carbon–hydrogen, and van der Waals bonds. Some of these dipole–dipole interactions, as well as hydrogen bonds, have the ability to modulate the physical properties of residues in the LacTv active site and therefore play important roles in the active conformation and stability of the protein–ligand complex (Salonen et al. 2011; Mehra et al. 2018).

Among the central amino acids involved in the LacTv-nonylphenol interaction, hydrophobic interactions via van der Waals forces occurred between Glu322-NP2EC, Gln262-4-NP, Gln262-NP1EO, Arg 443–4-NP, Ile 321–4-NP, Ile 321-NP2EC, Phe 259-NP2EC, and Phe 259-NP2EO. The contribution of this type of interaction is fundamental for the complexation of the ligand to the enzyme since the biological activity is related to the receptor–ligand affinity and the composition of the enzyme active sites is mainly composed of hydrophobic groups; therefore, this interaction plays an important role in the activity of laccase in the catalysis of substrate oxidation and reduction of molecular oxygen by maximizing contacts with specific residues of the enzyme receptor and influencing certain conformational modes of the enzyme so that the target molecule fits into the active site (Dunn 2010; Guimarães 2012).

#### LacTl-nonylphenol docking

**Fig. 7:**
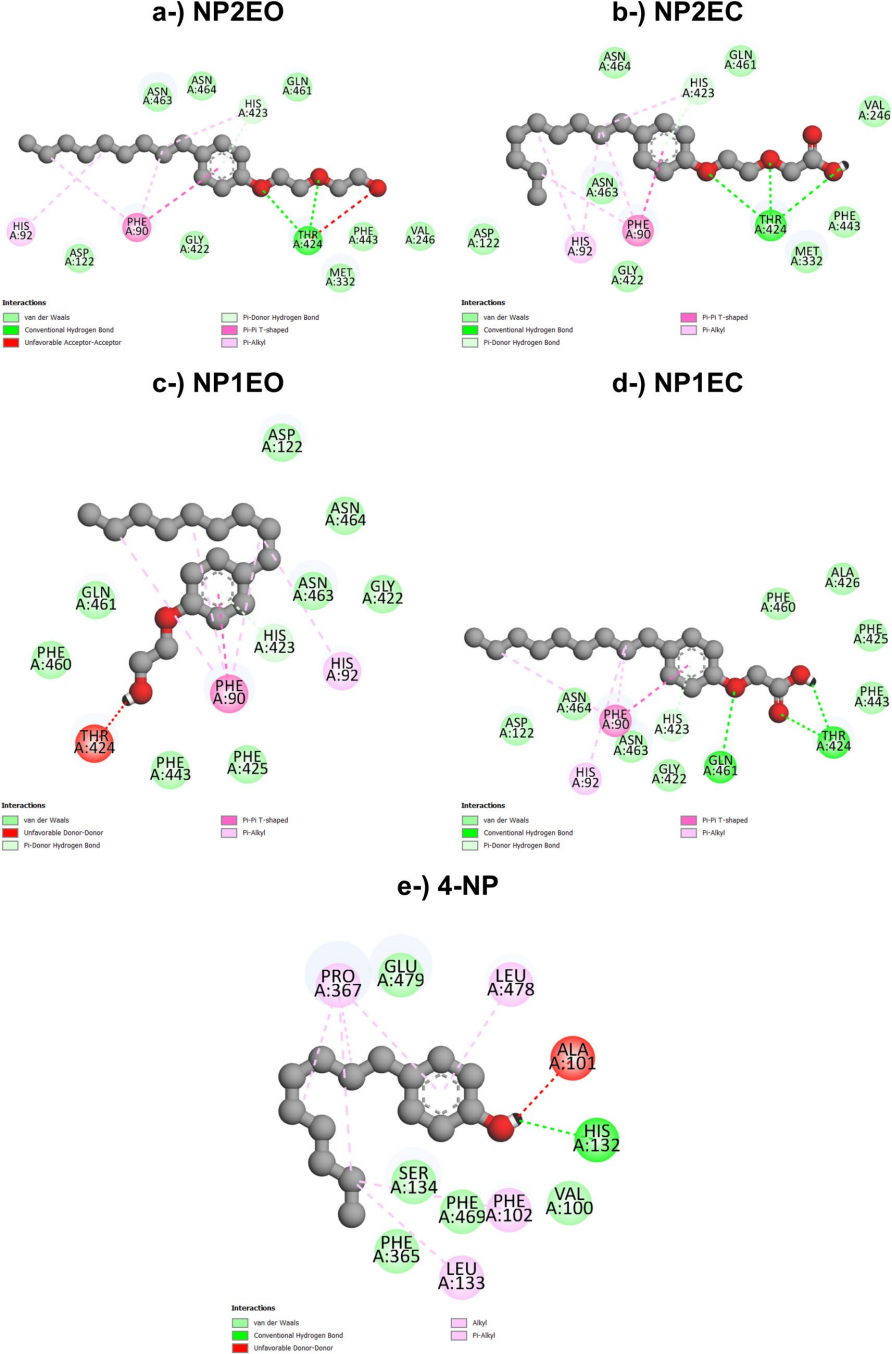
2D representations of the interactions between amino acid residues at the oxidative site of *T. lactinea* laccase (LacTl) with the compounds NP2EO (**a**) and NP1EO (**c**), observed during the degradation of nonylphenol ethoxylates under both aerobic and anaerobic conditions. Figures **b** and **d** illustrate the interactions of the oxidative site residues with NP2EC and NP1EC, respectively, which occur exclusively under aerobic conditions. Figure **e** shows the 2D interactions of the oxidative site residues of LacTv with 4-NP

LacTl interacted with nonylphenols through hydrogen bonds, π-donor hydrogen bonds, unfavorable donor–donor bonds, unfavorable acceptor–acceptor interactions, and T-shaped π–π, π–alkyl, alkyl and van der Waals interactions. Residues Ala101, His132, Phe102, Leu133, Pro367, Leu478, Thr424, Gln461, Phe90, His423, His92, and Phe90 were the central amino acids involved in the formation of the complexes. The LacTl–4-NP complex can be seen in Fig. 7e and Fig. 6b.

Like the interactions observed for the LacTv–4-NP complex discussed above, the amino acids Ala, Glu, Leu, Phe, Pro, Ser, and Val coincide with the formation of the laccase–4-NP complex for both species and contribute to the stability of the LacTl–4-NP complex through unfavorable donor‒donor interactions, π‒alkyl and alkyl bonds, and van der Waals interactions, demonstrating that the active site is conserved in both laccases.

As previously observed in the interaction of 4-NP with LacTv, amino acids from the LacTl active site also interact with the 4-NP alkyl chain through hydrophobic interactions of the alkyl and π-alkyl types. The results indicate that there are alkyl interactions between amino acids Phe102, Leu133, and Pro367 with the alkyl chain and π-alkyl interactions between the aromatic ring and residues Pro367 and Leu478, suggesting the involvement of these first three amino acids in the oxidation of the 4-NP alkyl chain and of residues Pro367 and Leu478 in the degradation of the final compound 3,4-dihydroxybenzoic acid by oxidative attack on the aromatic ring (Mtibaà et al. 2020).

The presence of hydrophilic uncharged, charged, and neutral amino acids such as Gln, His, and Thr in the LacTl active site, as well as the presence of amino acids of the same nature in the LacTv active site, contributes to the formation of hydrogen bonds and the occurrence of π-donor hydrogen bonds. They therefore play an important role in the interaction between laccase and the 4-NP intermediates, in which residues Thr424 and His423 form conventional hydrogen bonds and π-donor hydrogen bonds with NP2EO and NP1EO. The interaction with LacTl also revealed the presence of a hydrogen bond between residues His132, Thr424, and Gln461 and the ligands NP2EC, NP1EC, and 4-NP, and a π-donor hydrogen bond between His423 and the intermediates NP2EC and NP1EC. In addition, the presence of His residues also contributes to the stability of the complexes through T-shaped π‒π interactions with NP2EC and π‒alkyl interactions with NP2EO, NP2EC, NP1EO, and NP1EC.

#### Correlation of docking and theoretical descriptors

**Fig. 8:**
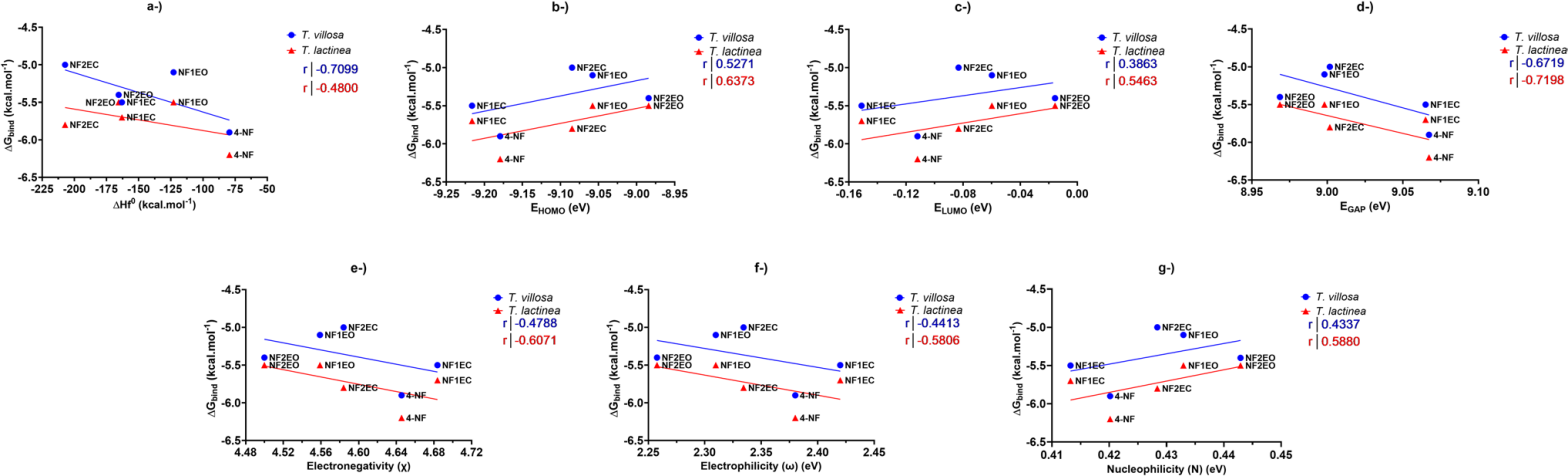
Correlation of global reactivity descriptors with Δ*G*_bind_ for the ligand–laccase complexes. Chemical potential (*μ*) was not included because it represents electronegativity with reversed sign, and electron affinity (*A*) and ionization potential (*I*) were omitted because they correspond to the negative of the LUMO and HOMO energies, respectively, making both parameters redundant in the analysis. Hardness and softness were not included, as both are functions of the energy gap and would reproduce the same trend

The analysis of the thermodynamic enthalpy (ΔHf^0^) and global reactivity descriptors derived from frontier molecular orbitals revealed consistent correlations between the ligand electronic properties and the binding affinity (Δ*G*_bind_) of the laccase–ligand complexes (Fig. 8). For LacTv, a strong negative correlation was observed between Δ*G*_bind_ and ΔHf^0^ (*r *= –0.7099) and the energy gap (GAP) (*r *= –0.6719). Moderate negative correlations were found for the ionization potential (*I*), electronegativity (*χ*), electrophilicity index (*ω*), and electron affinity (*A*) (–0.3863 ≥ *r *≥ –0.5271), whereas moderate positive correlations were observed for the EHOMO, chemical potential (*μ*), softness-related index (*σ*), and ELUMO (0.3863 ≤ *r* ≤ 0.5271).

For LacTl, GAP also showed a strong negative correlation with Δ*G*_bind_ (*r *= –0.7198), followed by strong correlations with ionization potential (I) (*r *= –0.6373) and electronegativity (χ) (*r *= –0.6071). EHOMO and μ showed strong positive correlations (0.6071 ≤ *r *≤ 0.6373). Moderate correlations were observed for electrophilicity (ω), electron affinity (A), and ΔHf^0^ (–0.5806 ≥ *r* ≥ –0.4800) and for ELUMO and softness-related index (σ) (0.5880 ≤ *r* ≤ 0.5463).

These trends indicate that ligands with a larger GAP correlate with more negative Δ*G*_bind_ values, meaning greater complex stability. In energetic terms, a higher GAP reflects a more electronically “rigid” molecule, with less available energy for electronic reorganization during the interaction, favoring a more stable enzyme-ligand complex (Hongyan et al. 2019; Qadir et al. 2025). This relationship is consistent with frontier orbital theory: EHOMO reflects the molecule’s ability to donate electrons, whereas ELUMO reflects its ability to accept electrons. Thus, differences in orbital energies modulate the electron density transfer between the ligand and the active site, influencing the strength and stability of the interaction (Oliveira 2015; Marinho et al. 2016).

However, none of the correlations was statistically significant (*p* > 0.05). Owing to the limited number of ligands (*n* = 5), these correlations should be interpreted as exploratory trends rather than definitive causal relationships.

Even so, the observed tendencies reinforce that electronic properties, particularly GAP, ionization potential, and electronegativity, may influence the stabilization of the enzyme‒ligand complex. Since these descriptors are sensitive to changes in protonation state (e.g., pH), experimental modulation of environmental conditions could further reduce Δ*G*_bind_ and increase complex affinity. The docking results complement experimental evidence that laccases can efficiently interact with phenolic endocrine disruptors (Nicolucci et al. 2011; Daâssi et al. 2016; Kimura et al. 2016; Hongyan et al. 2019), supporting the hypothesis that nonylphenols may undergo oxidative biotransformation via laccase-mediated mechanisms.

### Molecular dynamics

The average temperature of the system at equilibrium was 299.952 and 299.954 K (approximately 26.8 °C for both systems) during the simulation of the LacTv-4-NP and LacTl-4-NP complex interactions, respectively. The average pressure of the system at equilibrium remained close to 1.2 bar in both systems. The average density was 1052.250 and 1052.090 (kg/m^3^) for the LacTv-4-NP and LacTl-4-NP systems, respectively.

**Fig. 9:**
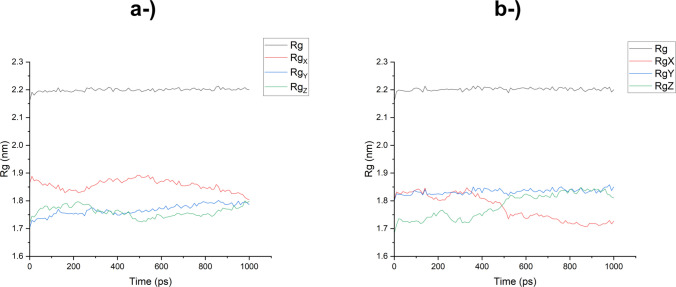
Radius of gyration (Rg) of the laccase from *T. villosa* (**a**) and from *T. lactinea* (**b**) plotted over time. The overall Rg (black line) as well as projections along the X (red), Y (blue), and Z (green) axes are shown

Rg (radius of gyration) analysis (Fig. 9) was performed to observe the degree of stability and compaction of the complexed protein during the simulation. The Rg values for both LacTv and LacTl around their own axes remained constant at approximately 2.2 nm, which shows that the two enzymes remained quite structurally stable without much variation during the dynamics, since the smaller the deviation from the initial position caused by the interactions established for the receptor–ligand complex, the stronger the evidence for the stability of the protein and that there is no tendency for it to denature in the presence of the contaminant (Arooj et al. 2020; Xue et al. 2023).

**Fig. 10:**
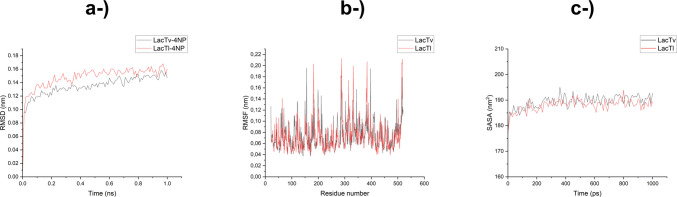
Structural stability analysis of *T. villosa* (black line) and *T. lactinea* (red) laccases during molecular dynamics simulations. **a** Root mean square deviation (RMSD) of backbone atoms for LacTv–4NP and LacTl–4NP complexes over time, showing the overall equilibration and stability of both systems. **b** Root mean square fluctuation (RMSF) per residue, indicating the flexibility profile of amino acid residues in LacTv and LacTl. **c** Solvent-accessible surface area (SASA) as a function of time for LacTv and LacTl, reflecting the surface dynamics and compactness of the enzyme structures throughout the simulation

The RMSD peaks of the complexes (Fig. 10a) were lower than 0.16 nm for LacTV-4-NP, with an average RMSD of 0.13 nm, and lower than 0.168 nm for LacTl-4-NP, with an average RMSD of 0.15 nm, demonstrating that complexation of laccase with 4-NP does not affect its stability, since low RMSD values indicate few structural changes in the protein during dynamics with respect to the initial position (Karunakaran and Muniyan 2023).

The RMSF (Fig. 10b) is similar to the RMSD, but it calculates the deviation for each residue during the simulation and analyzes the difference in the flexibility of the residues with respect to the initial position of the dynamics (Kumar et al. 2014). The highest fluctuations for amino acid residues were 0.21, 0.21, and 0.21 nm for residues Asn288, Asp517, and Gln384 in LacTl and 0.19, 0.19, and 0.19 nm for residues Arg156, His397, and Val288 in LacTv, respectively. This result indicates low fluctuation and further evidence that the system is in equilibrium and that the complexation of laccase with 4-NP and the flexibility of the residues do not affect the stability of the complexes, suggesting that there were no significant structural changes that could affect the interaction of the complex during the simulation (Sharma et al. 2020). The solvent-accessible surface area (SASA) (Fig. 10c) calculates the area of the protein that interacts with the solvent molecules, and a significant increase in this value during dynamics represents changes in the protein, such as denaturation or conformational changes induced by some interaction or by an increase in temperature (Mazola et al. 2015; Xue et al. 2023). The results obtained for the SASA of LacTv and LacTl were similar, with averages of 189.36 ± 2.59 and 188.01 ± 2.30 nm^2^, respectively. The variation was small, with SASA significantly stable, between 176.28 nm^2^ from the initial position to a maximum area of 195.04 nm^2^ during LacTv dynamics and between 177.35 and 193.88 nm^2^ during LacTl dynamics, with coefficients of variation of 1.36 and 1.22%, respectively. This suggests that there are no significant structural changes in the protein; therefore, the laccases of these two species are potential candidates for catalyzing the degradation of 4-NP in terms of bioremediation (Bhatt et al. 2021).

**Fig. 11:**
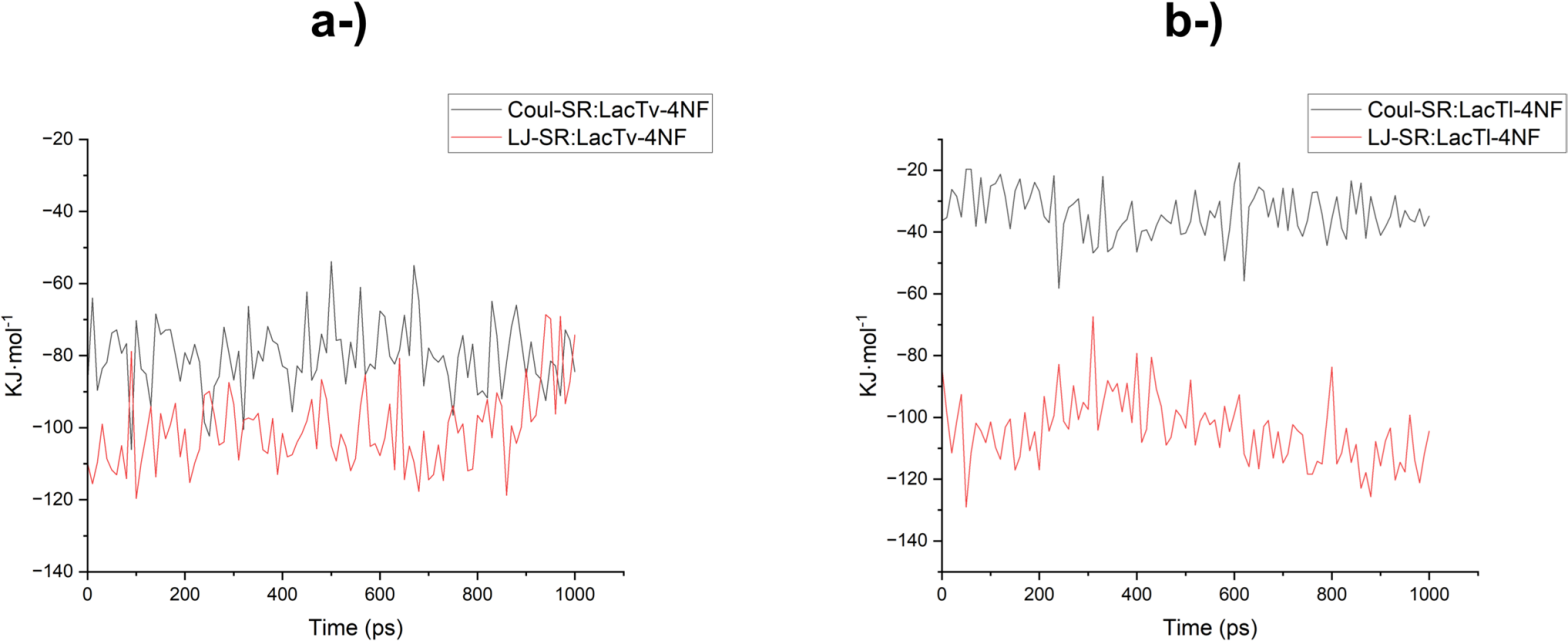
Electrostatic (Coul-SR) and van der Waals (LJ-SR) interaction energies between 4-NF and laccases during molecular dynamics simulations. **a** Interaction energies for the *T. villosa* laccase (LacTv–4NF) complex. **b** Interaction energies for the *T. lactinea* laccase (LacTl–4NF) complex. The plots illustrate the stability and relative contribution of nonbonded interactions throughout 1000 ps of simulation

The contribution of van der Waals/hydrophobic interaction energy (LJ-SR) to the stability of the laccase-4-NP complex was greater than the contribution of electrostatic interaction energy (Coul-SR) (Table 3 and Fig. 11), with LJ-SR energies of −100.79 and −104.15 kJ mol^−1^ and Coul-SR energies of −80.24 and −34.02 kJ mol^−1^, resulting in total interaction energies of −181.03 and −138.17 kJ mol^−1^ for the LacTv-4-NP and LacTl-4-NP complexes, respectively. The greater contribution of hydrophobic interactions can be explained by the increase in hydrophobicity of the products of ethoxylated nonylphenol during degradation (He et al. 2020; Ichale et al. 2023).

**Table 3:** Interaction energies of the LacTl-4NP and LacTv-4NP complexes

Energy	Average (kJ mol^−1^)		Err. Est. (kJ mol^−1^)	
	LacTv-4NP	LacTl-4NP	LacTv-4-NP	LacTl-4NP
Coul-SR	−80.2382	−34.0181	0.79	1.6
LJ-SR	−100.7920	−104.1550	2.5	3.3
Total	−181.0302	−138.1731	-	-

**Fig. 12:**
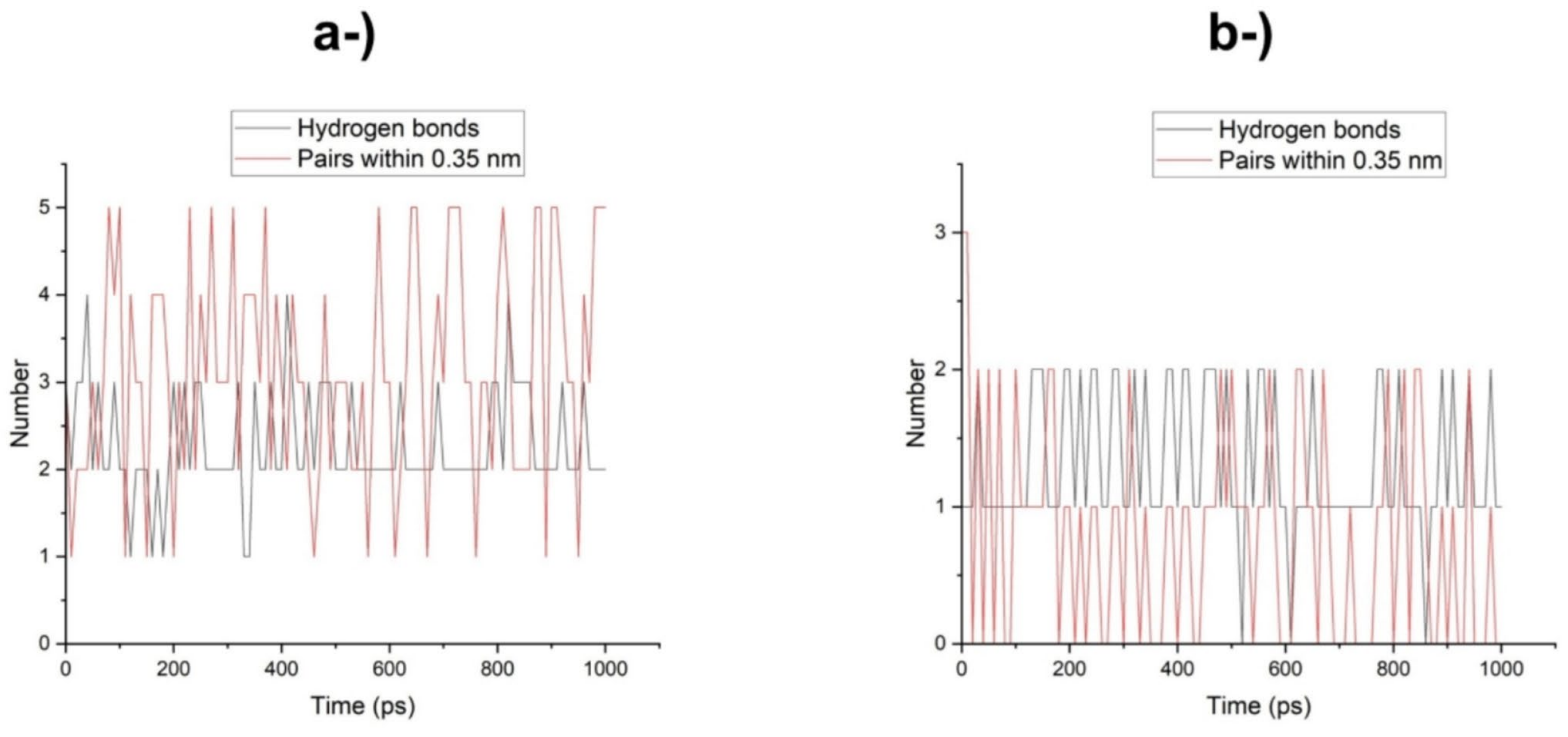
Hydrogen bond analysis between 4-NF and laccases during molecular dynamics simulations. **a** Number of hydrogen bonds and atom pairs within 0.35 nm for the *T. villosa* laccase (LacTv–4NF) complex. **b** Number of hydrogen bonds and atom pairs within 0.35 nm for the *T. lactinea* laccase (LacTl–4NF) complex. The plots show hydrogen bond fluctuations over 1000 ps of simulation, reflecting the stability of ligand–enzyme interactions

As shown in Fig. 12, the number of hydrogen bonds between the laccases during the dynamics varies from 1 to 5 for LacTv and from 0 to 3 for LacTl at the beginning of the simulation until it reaches a constant of 0–2 hydrogen bonds after 10 ps. Although the contribution of electrostatic interactions, such as hydrogen bonds, to the stability of the complexes was lower than that of hydrophobic interactions, as shown by the calculation of the interaction energies, the possibility of the occurrence of hydrogen bonds is fundamental for the catalytic activity of laccases, since hydrogen bonds tend to be linear and therefore have a strong ability to minimize the repulsion between the negative partial charges of the participating electronegative atoms, as well as being fundamental for maintaining the structural stability of the protein (Guimarães 2012; Barreiro et al. 2015).

The combination of docking and molecular dynamics techniques has been considered a powerful tool for obtaining information about the interactions between small molecules (ligands) and target proteins (Abdjan et al. 2023). The results obtained from this combination for the binding free energy (∆*G*_bind_) of the LacTv-4-NP and LacTl-4-NP complexes via molecular dynamics, which were calculated via the MM/PBSA package, revealed that the affinity of laccase for 4-NP increases when the reaction occurs in a solvated system (Table 4), resulting in average ∆*G*_bind_ values of −26.45 and −17.73 kcal·mol^−1^ for LacTv-4-NP and LacTl-4-NP, respectively.

**Table 4:** Decomposition of energies and total end-state binding free energy for the LacTl-4NP and LacTv-4NP complexes from equilibrated and stable MDS calculated by MM/PBSA (all values are expressed in kcal·mol^−1^)

Laccase-4-NP complex	LacTl	LacTv	LacTl	LacTv	LacTl	LacTv	LacTl	LacTv	LacTl	LacTv
ΔBOND	0	0	1.86	1.46	0	0	0.18	0.14	0	0
ΔANGLE	0	0	1.84	1.97	0	0	0.18	0.2	0	0
ΔDIHED	0	0	1.38	1.91	0	0	0.14	0.19	0	0
ΔUB	0	0	0.69	0.6	0	0	0.07	0.06	0	0
ΔIMP	0	0	0	0	0	0	0	0	0	0
ΔCMAP	0	0	0	0	0	0	0	0	0	0
ΔVDWAALS	−27.67	−26.79	0.28	1.12	2.59	2.59	0.03	0.11	0.26	0.26
ΔEEL	−4.89	−20.12	1.31	1.1	1.82	2.09	0.13	0.11	0.18	0.21
Δ1–4 VDW	0	0	1.21	1.23	0	0	0.12	0.12	0	0
Δ1–4 EEL	0	0	1.74	1.49	0	0	0.17	0.15	0	0
ΔEGB	19.01	24.75	0.01	0.33	1.42	1.46	0	0.03	0.14	0.15
ΔESURF	−4.18	−4.29	0.02	0.08	0.3	0.26	0	0.01	0.03	0.03
**ΔGGAS**	−**32.56**	−**46.91**	**1.51**	**1.68**	**2.66**	**2.5**	**0.15**	**0.17**	**0.26**	**0.25**
**ΔGSOLV**	**14.83**	**20.46**	**0.02**	**0.34**	**1.25**	**1.4**	**0**	**0.03**	**0.12**	**0.14**
**ΔTOTAL**	−**17.73**	−**26.45**	**1.51**	**1.71**	**2.43**	**2.24**	**0.15**	**0.17**	**0.24**	**0.22**

^a^SD(Prop.)—SD obtained with the propagation of the uncertainty formula

^b^SD—sample standard deviation

^c^SEM(Prop)—SEM obtained with the propagation of the uncertainty formula

^d^SEM—sample standard error of the mean

Therefore, since the accuracy of the binding free energy calculation methods used in this work is consistent with the results obtained experimentally and demonstrated by various studies (Hou et al. 2011; De Vivo et al. 2016; Wang et al. 2018), the results observed from the docking and molecular dynamics prediction analyses provide strong evidence, on the basis of the good binding affinity presented, that laccases from the *T. villosa* and *T. lactinea* species have strong potential to degrade 4-NP, as demonstrated experimentally for laccases from the *T. versicolor* species in the degradation of other phenolic disruptors, such as bisphenol A (Hongyan et al. 2019; Enyoh et al. 2022; Joshi et al. 2023).

## Conclusion

These data indicated the low reactivity of the molecules, reflecting the difficult degradation of these compounds. The results of this study demonstrate that laccases from *Trametes villosa* and *Trametes lactinea* strongly bind to the endocrine-disrupting compound 4-nonylphenol (4-NP) and its degradation intermediates. The combination of reactivity descriptors, molecular docking, and molecular dynamics revealed that 4-NP displays the highest binding affinity (Δ*G*_bind_ ≈ –6 kcal·mol^−1^ in docking) and that key residues in the oxidative active site, such as Ala, Glu, Leu, Phe, Pro, Ser, Val, and His, play a central role in stabilizing the enzyme‒substrate interaction through hydrogen bonding and hydrophobic contacts. These findings reinforce the potential role of laccases as catalytic agents in the oxidative degradation of persistent phenolic pollutants.

Molecular dynamics simulations confirmed the structural stability of both enzymes in the presence of 4-NP, with low RMSD and RMSF values and no significant changes in the radius of gyration or solvent-accessible surface area, indicating that the contaminant does not induce protein denaturation during complex formation. Additionally, MM/PBSA binding free energy calculations revealed even more favorable affinities in an explicitly solvated environment (Δ*G*_bind_ = −26.45 kcal·mol^−1^ for *T. villosa* and −17.73 kcal·mol^−1^ for *T. lactinea*), with hydrophobic interactions contributing predominantly to complex stabilization. These results demonstrate that the aqueous environment favors molecular recognition and stable complex formation between laccase and 4-NP.

Therefore, this work provides strong computational evidence that laccases from *T. villosa* and *T. lactinea* have high biocatalytic potential for the biodegradation of 4-nonylphenol, a persistent pollutant of major environmental concern. The identification of critical residues within the active site and the characterization of the forces that stabilize the enzyme–substrate complex offer valuable insights for future protein engineering efforts aiming to increase catalytic efficiency, as well as for designing in vitro assays and large-scale bioremediation strategies. Collectively, these results highlight the relevance of in silico tools as a strategic first step in selecting promising biocatalysts for the remediation of emerging contaminants. Thus, the results provide new perspectives for the study and application of laccases in the degradation of environmental pollutants, and for the continuation of this work, experimental studies are recommended to validate the theoretical results obtained via molecular simulation.

## Data Availability

The datasets generated and/or analyzed during the current study are available from the corresponding author upon reasonable request. The supplementary files include the LacTv and LacTl models, molecular docking data, molecular dynamics simulation results, and statistical analysis files: (i) SWISS-MODEL models of LacTv and LacTl, redocking files, full molecular docking files for all ligand‒enzyme complexes and PM7 mopac calculations of all ligands, (ii) complete molecular dynamics simulation energy output files and MM/PBSA calculations, and (iii) correlation matrix and statistical analysis files (GraphPad Prism).

## Code availability

The scripts used in this study (AutoDock Vina and GROMACS input scripts, MOPAC calculation scripts, and MM/PBSA configuration files) are available upon reasonable request to the corresponding author.
